# Factors associated with anemia among school-going adolescents aged 10–17 years in Zanzibar, Tanzania: a cross sectional study

**DOI:** 10.1186/s12889-023-16611-w

**Published:** 2023-09-18

**Authors:** Innocent Yusufu, Ilana R. Cliffer, Mashavu H. Yussuf, Cecilia Anthony, Frank Mapendo, Seif Abdulla, Mary Masanja, Amani Tinkasimile, Ali Salim Ali, Mary Mwanyika-Sando, Wafaie Fawzi

**Affiliations:** 1 Africa Academy for Public Health, Dar es Salaam, Tanzania; 2 Department of Global Health and Population, Harvard T.H. Chan School of Public Health, Harvard University, 90 Smith Street, 3rd Floor, Boston, MA United States of America; 3 Zanzibar Association for People Living with HIV/AIDS, Unguja, Zanzibar Tanzania

**Keywords:** Anemia, Adolescents, Micronutrient deficiency, Nutrition, Iron, Folic acid, Multiple micronutrients

## Abstract

**Background:**

Anemia among adolescents (ages 10–19 years) is a leading cause of morbidity and mortality in low- and middle-income countries and carries long-term health and economic consequences. To address the issue, policymakers and programmers require evidence of the burden of anemia among adolescents in specific contexts, as well as an understanding of the factors associated with anemia in this population.

**Methods:**

We conducted a cross-sectional survey as a baseline assessment to determine the prevalence and factors associated with anemia in secondary school students, as part of a cluster-randomized effectiveness trial testing different micronutrient supplementation strategies in addressing anemia among adolescents in Zanzibar. Between March 7th to 25th, 2022 the survey was conducted on 2,479 school-going adolescents aged 10–17 years from 42 schools on the island of Zanzibar, Tanzania. Hemoglobin concentration was measured along with the collection of socio-demographics, health, food frequency, and water, sanitation and hygiene data.

**Results:**

Based on the World Health Organization cutoffs for anemia, 53.3% of the sample had anemia (mild, moderate, or severe). Using chi-square tests and logistic regressions, we determined that females had higher odds of anemia than males (Adjusted OR = 1.47; 95% CI: 1.24, 1.74), those in the highest wealth quintile had lower odds of anemia than those in the lowest wealth quintile (Adjusted OR = 0.7; CI: 0.54, 0.91), stunted adolescents had higher odds of anemia than non-stunted students (Adjusted OR = 1.38; 95% CI: 1.06,1.81), and those who used shared toilets had higher odds of moderate or severe anemia than those with private toilet access (Adjusted OR = 1.68; CI: 1.07, 2.64).

**Conclusions:**

The high prevalence of anemia in this sample indicates an urgent need to address anemia among adolescents in Zanzibar, and the factors associated with anemia point to the importance of water, sanitation, and hygiene interventions in addition to dietary and nutritional support.

**Trial Registration:**

NCT05104554, registered 03/11/2021.

**Supplementary Information:**

The online version contains supplementary material available at 10.1186/s12889-023-16611-w.

## Background

An estimate of 1.3 billion adolescents between the ages of 10 and 19 years make up the global population, with more than 90% of them living in low and middle-income nations [1]. In 2019, the World Health Organization (WHO) reported 1.5 million adolescents died due to preventable or treatable causes. Nutrition and micronutrient deficiencies such as iron deficiency anemia continue to be among the leading cause of years lost in death and disability among adolescents [2]. Compared to other regions, Sub-Saharan Africa is expected to have more adolescents by 2050 making it an important area for adolescent health research and interventions [3]. Despite the burden and consequences of anemia among adolescents, this age group has been overlooked and under-studied [4].

The negative health impacts of anemia at such a crucial age in development can transcend to adulthood and lead to higher risks of maternal mortality and poor pregnancy outcome among girls [5, 6]. In Tanzania, 28% of adolescent girls become pregnant by age 18 years and 45% of these girls experience anemia during their first pregnancy [7]. Furthermore, the Tanzania Demographic and Health Survey TDHS 2015–2016 found that the prevalence of anemia was 45% among women aged 15–49 years in 2015, an increase from the rate recorded in 2010 (40%). Women in Zanzibar are even more likely to be anemic than women in Tanzania Mainland (60% versus 44%) [7]. While the prevalence rates of anemia among children under five years of age and women of reproductive age in Zanzibar are indeed high, it is important to note that the prevalence of anemia can vary among different population groups. Adolescents, being in a unique stage of development, may have different nutritional requirements and risk factors compared to children and women [4]. Therefore, it is possible that the prevalence of anemia among adolescents could be either higher or lower than the rates observed in the other two groups.

Anemia among adolescents is common possibly due to limited access to fruits, vegetables, and animal-source foods as a consequence of food insecurity [8]. Such nutritional deficiencies have serious consequences for the health and well-being of the adolescent population. In addition to nutritional causes, other causes of anemia include infections such as malaria and intestinal parasitic infection and chronic illness [9]. Despite nutritional deficiencies being the most common cause of anemia, subclinical deficiency make identification and treatment of anemia challenging. For example, depleted iron reserves may be presented long before symptoms of anemia present themselves [10].

As school attendance and performance are important determinants of career chances and long-term economic prospects for adolescents and their future families, addressing their nutritional needs is a crucial investment in their futures by improving their learning capacity. The school setting has been shown to be a cost-effective platform to address micronutrient deficiencies especially given economic losses from iron deficiency anemia (Shekar et al., 2017). However, in Zanzibar, there is lack of evidence to support the design and rollout of such intervention. We conducted a baseline survey to determine the burden of anemia and its determinants among adolescents in Zanzibar. This assessment is crucial for effective public health planning, intervention design, monitoring and evaluation, and improving health outcomes in this population. It provides valuable insights for policymakers and healthcare professionals to allocate resources, design targeted interventions, track progress, and address the specific needs and challenges of adolescents in reducing anemia rates and associated health risks.

This is in preparation of a cluster-randomized trial to determine the best school-based micronutrient supplementation program to prevent anemia. Findings from this study will provide an understanding of the prevalence of anemia and factors that are associated with it hence generate evidence for the design and rollout of interventions among adolescents.

## Methods

### Study design and setting

A school based cross-sectional study was conducted from March 7th to 25th 2022 in school-going adolescents aged 10–17 years. This was a baseline assessment prior to the rollout of a cluster-randomized study testing weekly iron and folic acid (IFA) against daily multiple micronutrient supplements (MMS) nutritional supplements in the management of anemia [11]. The study took place in Unguja, the largest island in Zanzibar, Tanzania. We selected 42 schools in urban or peri-urban areas (Wilaya ya Magharibi A and Wilaya ya Kati districts).

### Study participants

Adolescent girls and boys aged 10-17 years attending secondary schools were included in the study. We included public schools that have secondary students (form 1 (youngest) – form 4 (oldest)), and for which we had permission from school administrators. We randomized and enrolled 42 schools which were first matched on key characteristics (school rankings, number of students, and distance to main road), using coarsened exact matching (*cem* command, Stata) [12]. Once matched, schools were randomized to either the IFA, MMS, or control arms using a list randomizer (https://www.random.org/lists) [13]. One to two classes were randomly chosen within each school, with an aim to include a minimum of 50 eligible students per school and a maximum of 100 adolescents per school. Actual enrollments per school ranged from 20 to 108 students. We excluded schools with ongoing nutrition supplementation interventions, and adolescents with no informed consent from parents or self-reported pregnancy.

Sample sizes were not calculated for the present baseline analyses, only for the main study, as described previously [11]; however, we report minimum detectable effect sizes at the 5% alpha level with 80% power, for the factors potentially associated with anemia explored in this paper, in Supplementary Table 1.

### Data collection

Trained study staff collected data from adolescents at school, whose parents had provided consent and who had themselves provided assent to participate. Data collected included demographic and dietary surveys, and anthropometric measurements. A structured questionnaire comprising questions on socio-demographics, household possessions, physical activity (International Physical Activity Questionnaire for Adolescents), water, sanitation and hygiene practices (WASH), food frequency and preferences, and socio-emotional development (Strengths and Difficulties Questionnaire, Child Outcomes Research Consortium) was used to collect data.

In addition, a finger prick was used to assess hemoglobin levels and malaria status. Hemoglobin status was assessed using a portable battery-operated photometer (Hemocue 201 + machine) [14]. A capillary blood sample was taken by pricking the tip of the adolescents’ finger after rubbing it with a cotton swab immersed in alcohol. A 10 micro liter blood sample was collected with a sterile disposable lancet and the second drop was taken for hemoglobin measurement. The photometer was cleaned before every session. Hemoglobin levels were determined by certified laboratory technicians who were part of the research team. Rapid diagnostic test kits were used to screen the adolescents’ finger prick samples for malaria [15]. Results were read within one minute.

Anthropometric measurements (height and weight) were taken as double measurements, according to WHO standards. Height was measured using a Seca stadiometer (ShorrBoard, USA) and recorded to the nearest 0.1 cm. During the measurement, participants stood in the Frankfurt anatomical position. Shoes were taken off and prominent body parts (occipital, shoulder, buttocks and heel) touched the stadiometer. Weight was measured with the Seca 874 weighing scale (Seca, Germany) and recorded to the nearest 0.1 kg. Heavy clothes and shoes were taken off. Both the weighing scale and length board were calibrated daily.

The questionnaire was prepared in English and translated to Swahili and back translated to English to ensure consistency. It was piloted in two schools out of the selected schools. Twenty-four enumerators, three laboratory personnel, and one supervisor participated in the data collection. The data collection team received training for three days on the objectives and methodology of the study and the process of data collection by the principal investigator. Data collectors were supervised throughout the activity and daily meetings were held to monitor progress. Daily data cleaning and checks were done to check for accuracy.

### Variable specification

Adolescents’ anemia status was considered as the outcome variable and was defined according to WHO age- and sex- specific cutoffs (the gold standard for assessing anemia) as individual hemoglobin levels between 11 and 11.9 mg/dl, 8–10.9 g/dl, and lower than 8 g/dl as mild, moderate and severe respectively, for children 12–14 years and non-pregnant females over 15 years. For children 10–11 years old, mild anemia was defined as hemoglobin concentration between 11 and 11.4 g/dl and for males over 15, hemoglobin concentrations between 11.0 and 12.9 were considered mild anemia [16]. We investigated levels of anemia as binary outcome variables coded as having that level of anemia or not (any anemia vs. no anemia; moderate or severe anemia vs. no anemia), as well as a continuous outcome for hemoglobin level (g/dl).

Independent variables considered as potential factors associated with anemia in this study were identified by reviewing previous literature. Household possessions and assets were combined into a wealth status indicator using principal components analysis and then categorized into wealth quintiles.

Dietary data were collected through food frequency questionnaires in which the adolescents identified how many times in the last 30 days they had consumed each of a list of foods commonly found in Tanzania. We re-coded the food frequency data into number of times consumed per week, using the mid-point of the response categories (i.e., zero for “never or 1–3 times in the last month”, 3 for “2–4 times per week”, 5.5 for “5–6 times per week” and 7 for “once per day” or anything higher). For each food group of the Household Diet Diversity Score (HDDS) [17], we added up the weekly frequencies for all foods in that category. Weekly frequencies were then categorized into a three-level variable for “less than once per week”, “at least once per week” (1–6 times), or “daily” (at least 7 times per week). Iron-rich foods were of specific interest to investigate one-by-one, so we coded each of these as a binary variable for having been consumed at least once in the past month or not. Lastly, we coded an overall diet diversity indicator using the HDDS categories, by coding binary daily indicator variables for each category and adding them together to get a total score. The daily indicators were made by converting weekly frequencies to daily frequencies by dividing by 7 and assigning a “yes” to any food group category for which the total daily frequencies of foods in that category combined equaled at least 1, and a “no” if the total was less than 1. Thus, scores of HDDS ranged from 0 to 12, and were converted to a three-level variable for 0–3 food groups consumed, 4–5 groups consumed, or 6 + food groups consumed.

Variables for stunting (low height-for-age) and body mass index (BMI) category were coded by calculating anthropometric z-scores for height-for-age and BMI-for-age, using the 2007 WHO growth reference stata macro (who2007.ado) [18]. Stunting was considered height-for-age z-scores < -2 SD. For BMI z-scores and categorizations, BMI was first derived by dividing weight (kg) by height (m^2^). BMI z-scores were then calculated and categorized based on WHO guidelines for children and adolescents, into underweight (< -2 SD), normal weight (-2 SD to < 1 SD), and overweight/obese (1 SD to ≥ 2 SD). Overweight and obese categories were combined due to small frequencies of adolescents with obesity.

### Statistical analysis

To check for differences in anemia status by each of the independent variables, we first conducted descriptive analyses using chi-square tests and ran univariate logistic regressions. Based on the results of these univariate tests, we constructed a base adjusted model in which only socio-demographic and health variables that had been significantly associated with anemia in the univariate models at the 5% alpha level were included. In addition, we include age and malaria in these models despite statistical significance levels, as these factors were judged to be biologically important predictors of anemia, and potential confounders of the relationship between anemia and the other factors. The goal of these models was to understand the associations between each factor and anemia, while controlling for confounding of the other factors. We used this base adjusted model to determine adjusted relationships between each of the independent variables and anemia status by adding in each of the remaining independent variables one-by-one. We use logistic regression to obtain odds ratios for the relationship between the factors and each category of anemia (i.e. none vs. any, none vs. moderate/severe), and we use linear regression to model the relationship between the factors and continuous hemoglobin in g/dL. Analyses were done using Stata 16.1(Blackwell et al., 2009) (StataCorp, 2017. *Stata Statistical Software: Release 16*. College Station, TX: StataCorp LLC).

## Results

### Anemia prevalence

A total of 2,479 adolescents were enrolled across the 42 schools selected for the study. The prevalence of anemia among all adolescents in the sample was 1321(53.3%). Mild anemia was the most common 757 (30.5%), followed by moderate 546 (22.0%) and severe 18 (0.7%).

### Socio-demographic and health characteristics

Table 1 shows the baseline socio-demographic and health characteristics of the sample, overall and by anemia level. The majority of students enrolled were females 1516 (61.2%) between 12 and 14 years of age 1734 (69.9%). Around 1072 (75%) of females had experienced menarche at the time of the survey, 344 (14%) of students had a current cough, and77 (3%) were diagnosed with malaria using the rapid diagnostic tests administered during the survey. In addition, 248 (10%) of students were stunted, 127 (5%) were underweight, and 316 (13%) had overweight or obese BMI z-scores.

**Table 1:** Baseline socio-demographic characteristics of adolescents taking part in SAMIA trial, Zanzibar, 2022

	Overall (*N*=2,479)	No anemia (*n*=1,158)	Mild anemia (*n*=757)	Moderate/severe anemia (*n*=564)	***P***-value
**Age (*N*=2,479)**					
<12 years	15 (0.6)	5 (33.3)	7 (46.7)	3 (20.0)	0.002
12-14 years	1734 (69.9)	830 (47.9)	489 (28.2)	415 (23.9)	
>14 years	730 (29.4)	323 (44.2)	261 (35.8)	146 (20.0)	
**Sex (*N*=2,479)**					
Male	963 (38.8)	503 (52.2)	296 (30.7)	164 (17.0)	<0.001
Female	1516 (61.2)	655 (43.2)	461 (30.4)	400 (26.4)	
**Currently lives with (*N*=2,479)**					
Mother	395 (15.9)	171 (43.3)	125 (31.6)	99 (25.1)	0.733
Father	78 (3.1)	32 (41.0)	30 (38.5)	16 (20.5)	
Other male guardian	69 (2.8)	32 (46.4)	23 (33.3)	14 (20.3)	
Other female guardian	265 (10.7)	133 (50.2)	75 (28.3)	57 (21.5)	
Sibling(s)	55 (2.2)	29 (52.7)	14 (25.5)	12 (21.8)	
Both Father and Mother	1483 (59.8)	703 (47.4)	443 (29.9)	337 (22.7)	
Other, specify:	134 (5.4)	58 (43.3)	47 (35.1)	29 (21.6)	
**Number of siblings (*N*=2,473)**					
0-4 siblings	1652 (66.8)	774 (46.9)	512 (31.0)	366 (22.2)	0.818
5-9 siblings	754 (30.5)	350 (46.4)	221 (29.3)	183 (24.3)	
>9 siblings	67 (2.7)	32 (47.8)	20 (29.9)	15 (22.4)	
**Father's occupation (*N*=1,630)**					
Farmer	395 (24.2)	179 (45.3)	123 (31.1)	93 (23.5)	0.637
Livestock keeper	17 (1)	6 (35.3)	6 (35.3)	5 (29.4)	
Merchant	354 (21.7)	170 (48.0)	99 (28.0)	85 (24.0)	
Teacher	67 (4.1)	32 (47.8)	25 (37.3)	10 (14.9)	
Government Worker	263 (16.1)	140 (53.2)	73 (27.8)	50 (19.0)	
Unemployed	93 (5.7)	40 (43.0)	33 (35.5)	20 (21.5)	
Other, specify	415 (25.5)	188 (45.3)	128 (30.8)	99 (23.9)	
Don't know	26 (1.6)	12 (46.2)	9 (34.6)	5 (19.2)	
**Mother's occupation (*N*=2,142)**					
Farmer	434 (20.3)	202 (46.5)	120 (27.6)	112 (25.8)	0.682
Livestock keeper	12 (0.6)	2 (16.7)	4 (33.3)	6 (50.0)	
Merchant	723 (33.8)	337 (46.6)	223 (30.8)	163 (22.5)	
Teacher	155 (7.2)	73 (47.1)	49 (31.6)	33 (21.3)	
Other Government Worker	83 (3.9)	38 (45.8)	27 (32.5)	18 (21.7)	
Unemployed	214 (10)	103 (48.1)	67 (31.3)	44 (20.6)	
Homemaker	434 (20.3)	206 (47.5)	126 (29.0)	102 (23.5)	
Other, specify:	72 (3.4)	38 (52.8)	22 (30.6)	12 (16.7)	
Don't know	15 (0.7)	8 (53.3)	5 (33.3)	2 (13.3)	
**Father's education (*N*=1,630)**					
None	77 (4.7)	35 (45.5)	22 (28.6)	20 (26.0)	0.447
Primary	259 (15.9)	115 (44.4)	82 (31.7)	62 (23.9)	
Secondary	458 (28.1)	213 (46.5)	156 (34.1)	89 (19.4)	
Technical/Vocational	18 (1.1)	8 (44.4)	4 (22.2)	6 (33.3)	
University/College	104 (6.4)	56 (53.8)	28 (26.9)	20 (19.2)	
Don't know	714 (43.8)	340 (47.6)	204 (28.6)	170 (23.8)	
**Mother's education (*N*=2,141)**					
None	157 (7.3)	71 (45.2)	51 (32.5)	35 (22.3)	0.65
Primary	466 (21.8)	218 (46.8)	134 (26.8)	114 (24.5)	
Secondary	610 (28.5)	295 (48.4)	177 (29.0)	138 (22.6)	
Technical/Vocational	18 (0.8)	12 (66.7)	5 (27.8)	1 (5.6)	
University/College	117 (5.5)	57 (48.7)	39 (33.3)	21 (17.9)	
Don’t know	773 (36.1)	354 (45.8)	236 (30.5)	183 (23.7)	
**Wealth Quintile (*N*=2,479)**					
1	500 (20.2)	207 (41.4)	160 (32.0)	133 (26.6)	0.002
2	505 (20.4)	236 (46.7)	154 (30.5)	115 (22.8)	
3	489 (19.7)	216 (44.2)	139 (28.4)	134 (27.4)	
4	514 (20.7)	255 (49.6)	161 (31.3)	98 (19.1)	
5	471 (19)	244 (51.8)	143 (30.4)	84 (17.8)	
**Seen menstruation (*N*=1,416)**					
No	344 (24.3)	166 (48.3)	99 (28.8)	79 (23.0)	0.116
Yes	1072 (75.7)	452 (42.2)	328 (30.6)	292 (27.2)	
**Home garden available (*N*=2,463)**					
No	1415 (57.5)	647 (45.7)	431 (30.5)	337 (23.8)	0.325
Yes	1048 (42.5)	503 (48.0)	321 (30.6)	244 (23.3)	
**Current cough (*N*=2,339)**					
No	2135 (86.1)	983 (46.0)	653 (30.6)	499 (23.4)	0.129
yes	344 (13.9)	175 (50.9)	104 (30.2)	65 (18.9)	
**Malaria diagnosis**					
No	2402 (96.9)	1120 (46.6)	735 (30.6)	547 (22.8)	0.889
Yes	77 (3.1)	38 (49.4)	22 (28.6)	17 (22.1)	
**Height for age z-score category (*N*=2467)**					
Normal	2219 (89.9)	1053 (47.5)	671 (30.2)	495 (22.3)	0.051
Stunted	248 (10.1)	98 (39.5)	83 (33.5)	67 (27.0)	
**BMI for age z-score category (*N*=2,464)**					
Underweight	127 (5.2)	67 (52.8)	41 (32.3)	19 (15.0)	0.147
Normal weight	2021 (82)	947 (46.9)	608 (30.1)	466 (23.1)	
Overweight/obese	316 (12.8)	135 (42.7)	105 (33.2)	76 (24.1)	

Values are n(%). *P*-values derived from Chi-square test or Fisher's exact test if the cell count <5, Fisher's exact test performed

### Dietary characteristics

Dietary characteristics of the sample are shown in Table 2. Less than half of the overall sample consumed at least 6 food groups 1138 (45.9%). Most people consumed cereals and vegetables daily 2053 (82.8%), 1891 (76.3%) respectively, while daily consumption of legumes 842 (34%), 884 fruits (37.8%), fish 1392 (56.2%), milk 1447 (58.4%), and tubers 1153 (46.5%) was less common. Few people consumed meat 187 (7.5%) or eggs 47 (1.9%) daily. In terms of specifically iron-rich foods (Table 3), we display the prevalence of those who consumed the food at least once in the last month. The most commonly consumed iron-rich food was fried fish 2266 (91.4%), followed by fresh fish 2104 (84.9%), chicken 1864 (75.2%), sardines 1702 (68.7%), and beef 1258 (50.7%).

**Table 2:** Baseline dietary characteristics of adolescents taking part in SAMIA trial, Zanzibar, 2022

	Overall (*N*=2,479)	No anemia (*n*=1,158 )	Mild anemia (*n*=757)	Moderate/severe anemia (*n*=564)	***P***-value
**Household diet diversity score (*N*=2,479)**					
3 and less	574 (23.2)	259(45.1)	178 (31)	137 (23.9)	0.486
4 to 5	767 (30.9)	45 (24.1)	241 (31.4)	181 (23.6)	
6 and more	1138 (45.9)	554 (48.7)	338 (29.7)	246 (21.6)	
**Food consumption (*N*=2,479)**					
**Cereals (***N***=2,479)**					
< 1 per week	56(2.3)	27(48.2)	16(28.6)	13(23.2)	0.191
1-6 per week	370(14.9)	154(41.6)	116(31.4)	100(27)	
Daily	2053(82.8)	977(47.6)	625(30.4)	451(22)	
**Vegetables (*N*=2,279)**					
< 1 per week	196(7.9)	72(36.7)	66(35.9)	43(21.9)	0.321
1-6 per week	392(15.8)	124(31.6)	386(29.5)	86(21.9)	
Daily	1891(76.3)	561(29.7)	256(30.3)	435(23)	
**Legumes (*N*=2,479)**					
< 1 per week	657(26.5)	297(45.2)	211(32.1)	149(22.7)	0.851
1-6 per week	980(39.5)	462(47.1)	291(29.7)	227(23.2)	
Daily	842(34)	399(47.4)	255(30.3)	188(22.3)	
**Fruits (*N*=2,479)**					
< 1 per week	202(8.6)	90(40.9)	73(33.2)	57(25.9)	0.359
1-6 per week	1253(53.6)	174(45)	122(31.5)	91(23.5)	
Daily	884(37.8)	894(47.8)	562(30)	416(22.2)	
**Meat (*N*=2,479)**					
< 1 per week	1378(55.6)	631(45.8)	424(30.8)	323(23.4)	0.248
1-6 per week	914(36.9)	438(47.9)	267(29.2)	209(22.9)	
Daily	187(7.5)	89(47.6)	66(35.3)	32(17.1)	
**Eggs (*N*=2,479)**					
< 1 per week	1938(78.2)	886(45.7)	598(30.9)	454(23.4)	0.217
1-6 per week	494(19.9)	244(49.4)	148(30)	102(20.6)	
Daily	47(1.9)	28(59.6)	11(23.4)	8(17)	
**Fish (*N*=2,479)**					
< 1 per week	230(9.3)	98(42.6)	72(31.3)	60(26.1)	0.178
1-6 per week	857(34.6)	382(44.6)	268(31.3)	207(24.2)	
Daily	1392(56.2)	678(48.7)	417(30)	297(21.3)	
**Milk (*N*=2,479)**					
< 1 per week	280(11.3)	125(44.6)	85(30.4)	70(25)	0.74
1-6 per week	752(30.3)	353(46.9)	222(29.5)	177(23.5)	
Daily	1447(58.4)	680(47)	450(31.1)	317(21.9)	
**Fats (*N*=2,479)**					
< 1 per week	1938(78.2)	886(45.7)	598(30.9)	454(23.4)	0.217
1-6 per week	494(19.9)	244(49.4)	148(30)	102(20.6)	
Daily	47(1.9)	28(59.6)	11(23.4)	8(17)	
**Beverages (*N*=2,479)**					
< 1 per week	234(9.4)	94(40.2)	74(31.6)	66(28.2)	0.172
1-6 per week	740(29.9)	347(46.9)	222(30)	171(23.1)	
Daily	1505(60.7)	717(47.6)	461(30.6)	327(21.7)	
**Sweets (*N*=2,479)**					
< 1 per week	1376(55.5)	639(46.4)	428(31.1)	309(22.5)	0.954
1-6 per week	964(38.9)	454(47.1)	289(30)	221(22.9)	
Daily	139(5.6)	65(46.8)	40(28.8)	34(24.5)	
**Tubers(*N*=2,479)**					
< 1 per week	315(12.7)	137(43.5)	97(30.8)	81(25.7)	0.432
1-6 per week	1011(40.8)	470(46.5)	303(30)	238(23.5)	
Daily	1153(46.5)	551(47.8)	357(31)	245(21.2)	

Values are n(%) or mean SD±. *P*-values derived from Chi-square test or Fisher's exact test if the cell count <5, Fisher's exact test performed. Data derived from food frequency questionnaire in which the reference period for consumption was the last 30 days

**Table 3:** Consumption of iron-rich foods among adolescents taking part in SAMIA trial, Zanzibar, 2022

	Overall (*N*= 2,479)	No anemia (*n*=1,158)	Mild anemia (*n*=757)	Moderate/severe anemia (*n*=564)	***P***-value
**Tamarind (*N*=2,479)**					
No	2071(83.5)	963(46.5)	636(30.7)	472(22.8)	0.88
Yes	408(16.5)	195(47.8)	121(29.7)	92(22.5)	
**Beans (*N*=2,479)**					
No	1795(72.4)	832(46.4)	545(30.4)	418(23.3)	0.586
Yes	684(27.6)	326(47.7)	212(31.0)	146(21.3)	
**Spinach (*N*=2,479)**					
No	2115(85.3)	1009(47.7)	628(29.7)	478(22.6)	0.038
Yes	364(14.7)	149(40.9)	129(35.4)	86(23.6)	
**Pumpkin leaves (*N*=2,479)**					
No	2032(82)	947(46.6)	616(30.3)	469(23.1)	0.688
Yes	447(18)	211(47.2)	141(31.5)	95(21.3)	
**Pumpkin (*N*=2,479)**					
No	1677(67.6)	798(47.6)	488 (29.1)	380(22.7)	0.387
Yes	802(32.4)	360(44.9)	220 (27.4)	184(22.9)	
**Beef (*N*=2,479)**					
No	1221(49.3)	562(46.0)	366(30.0)	293(24.0)	0.345
Yes	1258(50.7)	596(47.4)	391(31.1)	271(21.5)	
**Liver (*N*=2,479)**					
No	2020(81.5)	925(45.8)	617(30.5)	478(23.7)	0.051
Yes	459(18.5)	233(50.8)	140(30.5)	86(18.7)	
**Chicken (*N*=2,479)**					
No	615(24.8)	267(43.4)	196(31.9)	152(24.7)	0.152
Yes	1864 (75.2)	891(47.8)	561(30.1)	412(22.1)	
**Fried fish (*N*=2,479)**					
No	213(8.6)	90(42.3)	74(34.7)	49(23.0)	0.309
Yes	2266(91.4)	1068(47.1)	683(30.1)	515(22.7)	
**Fresh fish (*N*=2,479)**					
No	375(15.1)	173(46.1)	106(28.3)	96(25.6)	0.308
Yes	2104(84.9)	985(46.8)	651(30.9)	468(22.2)	
**Sardines (*N*=2,479)**					
No	777(31.3)	343(44.1)	244(31.4)	190(24.5)	0.19
Yes	1702(68.7)	815(47.9)	513(30.1)	374(22.0)	
**Dried Fish (*N*=2,479)**					
No	1730(69.8)	810(46.8)	530(30.6)	390(22.5)	0.932
Yes	749(30.2)	348(46.5)	227(30.3)	174(23.2)	

Values are n(%). *P*-values derived from Chi-square test. Data derived from food frequency questionnaire in which the reference period for consumption was the last 30 days. The value label "Yes" corresponds to consumption of the food at least once within the past month. Other iron-rich foods examined include pork and green beans; however, these were excluded from the table due to 99-100% of the sample not consuming them

### WASH characteristics

Table 4 displays the water, sanitation, and hygiene characteristics and practices of adolescents in the sample. About 2022 (80%) of the sample had water piped either directly into their houses or into their neighborhoods, while 449 (18%) drank well or surface water. Roughly a quarter of adolescents 594 (24.1%) reported that their households treated their water, and the most common treatment technique was boiling 319 (12.9%). A majority of participants had flush or pour toilets at home 1666 (67.3%) and reported brushing their teeth at least twice a day 1568 (63.3%). Slightly less than half of participants reported always washing their hands before eating 1182 (47.7%), and even fewer 987 (39.8%) reported always washing their hands after using the toilet.

**Table 4:** Water, sanitation, and hygiene characteristics of adolescents taking part in SAMIA trial, Zanzibar, 2022

	**Overall (*N*= 2,479)**	**No anemia (*n*=1,158)**	**Mild anemia (*n*=757)**	**Moderate/severe anemia (*n*=564)**	***P*-value**
**Water Source (*N*=2471)**					
Piped into house /bottled water	1457(59)	687(47.2)	441(30.3)	329(22.6)	0.952
Piped into neighborhood	565(22.9)	262(46.4)	170(30.1)	133(23.5)	
Well or surface water	449(18.2)	205(45.7)	143(31.8)	101(22.5)	
**Household treats water (*N*=2464)**					
No	1870(75.9)	867(46.4)	570(30.5)	433(23.2)	0.628
Yes	594(24.1)	286(48.1)	181(30.5)	127(21.4)	
**Water Treatment method (*N*=2479)**					
Boil	319(12.9)	139(43.6)	104(32.6)	76(23.8)	0.312
Add bleach/chlorine	220(8.9)	117(53.2)	61(27.7)	42(19.1)	
Do not treat water	1885(76)	872(46.3)	576(30.6)	437(23.2)	
Other, specify	55(2.2)	30(54.5)	16(29.1)	9(16.4)	
**Toilet type(*N*=2476)**					
Flush or pour flush toilet	1666(67.3)	790(47.4)	517(31.0)	359(21.5)	0.111
Pit toilet/latrine/no facility/bush/filed	810(32.7)	368(45.4)	237(29.3)	205(25.3)	
**Shared toilet (*N*=2462)**					
No	2345 (95.2)	1102 (47.0)	722 (30.8)	521 (22.2)	0.056
Yes	117 (4.8)	50 (42.7)	30 (25.6)	37 (31.6)	
**Brushing teeth (*N*=2479)**					
No	19 (0.8)	11 (57.9)	4 (21.1)	4 (21.1)	0.672
Once per day	892 (36)	417 (46.7)	282 (31.6)	193 (21.6)	
Twice or more per day	1568 (63.3)	730 (46.6)	471 (30.0)	367 (23.4)	
**Dentist visits in the past year (*N*=2479)**					
No	2253 (90.9)	1059 (47.0)	681 (30.2)	513 (22.8)	0.54
Yes	226 (9.1)	99 (43.8)	76 (33.6)	51 (22.6)	
**Handwashing before eating(*N*=2479)**					
Never/Rarely	230(9.3)	113 (49.1)	76 (33.0)	41 (17.8)	0.247
Sometimes	318 (12.8)	162 (50.9)	84 (26.4)	72 (22.6)	
Most of the time	749 (30.2)	340 (45.4)	240 (32.0)	169 (22.6)	
Always	1182 (47.7)	543 (45.9)	357 (30.2)	282 (23.9)	
**Handwashing after toilet (*N*=2467)**					
Never/Rarely	415 (16.7)	201 (48.4)	129 (31.1)	85 (20.5)	0.269
Sometimes	472 (19)	216 (45.8)	160 (33.9)	96 (20.3)	
Most of the time	605 (24.4)	293 (48.4)	170 (28.1)	142 (23.1)	
Always	987 (39.8)	448 (45.4)	298 (30.2)	241 (24.4)	
**Handwashing method (*N*=2467)**					
Proper	1801(73)	837 (46.5)	558 (31.0)	406 (22.5)	0.864
Improper	666(27)	313 (47.0)	199 (29.9)	154 (23.1)	

Values are n(%). *P*-values derived from Chi-square test or Fisher's exact test if the cell count <5, Fisher's exact test performed. Data derived from food frequency questionnaire in which the reference period for consumption was the last 30 days. Proper handwashing was defined as using soap and water. Any other method (including only water) was considered improper

### Relationships between socio-demographic, dietary, and WASH factors and anemia

Anemia was related to several socio-demographic and health variables, as well as some factors related to water and sanitation (Table 5). Females had higher odds of any anemia (aOR = 1.47; 95% CI: 1.24, 1.74) and moderate/severe anemia (aOR = 1.87; 95% CI: 1.51,2.32) compared to males. The hemoglobin levels among females were also lower compared to males (b=-062; 95% CI: -0.73, -0.51). Wealth index was found to be associated with anemia. Adolescents who were found in the fourth wealth quintile had lower odds of any anemia (aOR = 0.72; CI: 0.56,0.92) and moderate/severe anemia (aOR = 0.60; CI: 0.43,0.82) compared to the lowest quintile in both the crude and adjusted models. Similar relationship was seen between the fifth quintile and the lowest quintile (aOR = 0.7; CI: 0.54, 0.91)), and hemoglobin level was also found to be lower in both the fourth (b = 0.35; CI:0.17,0.53) and fifth (b = 0.30; CI:0.12,0.47) wealth quintile compared to the first quintile.

**Table 5:** Regression of sociodemographic, food groups and WASH variables on anemia status among adolescents taking part in SAMIA trial, 2021-2022

	Anemia (Y/N)		Anemia (None vs Mod/Severe)		Hemoglobin (g/dl)	
	Crude OR (95% CI)	Adjusted OR (95% CI)	Crude OR (95% CI)	Adjusted OR (95% CI)	Crude OR (95% CI)	Adjusted OR (95% CI)
**Sex (Ref=Male)**						
Female	1.44***(1.22-1.69)	1.47***(1.24-1.74)	1.87***(1.51-2.32)	1.83***(1.46-2.29)	-0.62***(-0.73--0.51)	-0.56***(-0.67--0.44)
**Age (Ref= < 12)**						
12-14	0.54(0.19-1.6)	0.51(0.17-1.51)	0.84 (0.2-3.54)	0.8(0.18-3.44)	0.62(-0.53- 1.76)	0.67(-0.03-1.37)
>14	0.63(0.21-1.86)	0.62(0.21-1.86)	0.73 (0.17-3.09)	0.75(0.17-3.29)	1.15 (-0.01-2.30)	0.97(0.27-1.68)
**Wealth Quintile (Ref=1)**						
2	0.81(0.63-1.03)	0.81(0.63-1.05)	0.76(0.56-1.04)	0.76(0.55-1.04)	0.1(-0.07-0.28)	0.09(-0.08-0.26)
3	0.89(0.69-1.15)	0.93(0.72-1.19)	0.97(0.71-1.31)	0.98(0.72-1.34)	0.03(-0.15-0.2)	0.05(-0.13-0.22)
4	0.72**(0.56-0.92)	0.75*(0.58-0.96)	0.60**(0.43-0.82)	0.6**(0.43-0.83)	0.24**(0.06-0.41)	0.24**(0.07-0.42)
5	0.66**(0.51-0.85)	0.70**(0.54-0.91)	0.54***(0.39-0.75)	0.58**(0.41-0.81)	0.35***(0.17-0.53)	0.30**(0.12-0.47)
**Malaria diagnosis (Ref=No)**						
Yes	0.9(0.57-1.41)	0.92(0.58-1.46)	0.92(0.51-1.64)	0.99(0.54-1.8)	0(-0.33-0.32)	-0.08(-0.58-0.42)
**Stunting (Ref=Not stunted)**						
Stunted	1.38*(1.06-1.81)	1.50**(1.13-1.97)	1.45*(1.05-2.02)	1.7**(1.21-2.39)	-0.14(-0.33-0.04)	-0.44**(-0.74--0.15)
**BMI for age z-score category (Ref=Normal)**						
Underweight	0.79(0.55-1.13)	0.77(0.53-1.11)	0.58*(0.34-0.97)	0.56*(0.32-0.95)	0.19(-0.06-0.45)	0.83(0.43-1.23)
Overweight/obese	1.18(0.93-1.5)	1.17(0.92-1.49)	1.14(0.85-1.55)	1.07(0.79-1.46)	-0.18(-0.35--0.01)	-0.07(-0.33-0.2)
**Number of siblings (Ref= 0-4)**						
5-9	1.02(0.86-1.21)	1.06(0.88-1.26)	1.11(0.89-1.37)	1.2(0.96-1.51)	-0.1(-0.22-0.03)	-0.14*(-0.26--0.02)
>9	0.96(0.59-1.57)	0.91(0.55-1.49)	0.99(0.53-1.85)	1.01(0.53-1.93)	-0.01(-0.36-0.33)	-0.06(-0.39-0.28)
**Currently live with (Ref=Mother)**						
Father	1.1(0.67-1.8)	1.24(0.75-2.04)	0.86(0.45-1.65)	1.03(0.53-2.02)	-0.03(-0.38-0.31)	-0.17(-0.51-0.17)
Other male guardian	0.88(0.53-1.47)	0.84(0.5-1.41)	0.76(0.38-1.48)	0.71(0.36-1.42)	0.23(-0.13-0.59)	0.21(-0.15-0.56)
Other female guardian	0.76(0.55-1.04)	0.73(0.53-1.01)	0.74(0.5-1.1)	0.69(0.46-1.04)	0.09(-0.13-0.31)	0.14(-0.07-0.36)
Sibling(s)	0.68(0.39-1.2)	0.65(0.36-1.14)	0.71(0.35-1.46)	0.69(0.33-1.44)	0.14(-0.26-0.54)	0.13(-0.26-0.52)
Both Father and Mother	0.85(0.68-1.06)	0.92(0.73-1.16)	0.83(0.63-1.1)	0.9(0.68-1.21)	0.09(-0.07-0.24)	0.02(-0.13-0.18)
Other, specify:	1(0.67-1.49)	0.97(0.65-1.44)	0.86(0.52-1.44)	0.8(0.48-1.35)	-0.02(-0.3-0.26)	0.03(-0.25-0.3)
**Father's occupation (Ref=Farmer)**						
Livestock keeper	1.52(0.55-4.19)	1.57(0.56-4.41)	1.6(0.48-5.39)	1.68(0.48-5.83)	-0.3(-0.98-0.39)	-0.26(-0.94-0.41)
Merchant	0.9(0.67-1.2)	0.93(0.69-1.25)	0.96(0.67-1.38)	1.06(0.73-1.55)	-0.01(-0.21-0.2)	-0.02(-0.22-0.19)
Teacher	0.91(0.54-1.52)	0.95(0.56-1.62)	0.6(0.28-1.28)	0.71(0.33-1.55)	0.24(-0.13-0.6)	0.26(-0.1-0.62)
Government Worker	0.73(0.53-1)	0.83(0.6-1.16)	0.69(0.46-1.03)	0.9(0.58-1.39)	0.21(-0.01-0.43)	0.1(-0.13-0.32)
Unemployed	1.1(0.7-1.73)	1.12(0.7-1.78)	0.96(0.53-1.74)	0.99(0.54-1.83)	-0.04(-0.36-0.28)	-0.02(-0.34-0.29)
Other, specify	1(0.76-1.32)	1.05(0.79-1.4)	1.01(0.71-1.44)	1.11(0.77-1.6)	-0.05(-0.24-0.15)	-0.07(-0.26-0.13)
Don't know	0.97(0.44-2.14)	0.99(0.44-2.24)	0.8(0.27-2.34)	0.71(0.23-2.21)	-0.08(-0.64-0.48)	0(-0.55-0.55)
**Mother occupation (Ref=Farmer)**						
Livestock keeper	4.35(0.94-20.1)	5.34(1.14-24.94)	5.41(1.07-27.26)	6.22(1.2-32.2)	-1.02(-1.83--0.22)	-1.1(-1.89--0.31)
Merchant	1(0.79-1.27)	1.09(0.85-1.4)	0.87(0.65-1.17)	0.93(0.68-1.26)	0.04(-0.13-0.21)	0.01(-0.15-0.18)
Teacher	0.98(0.68-1.41)	1.15(0.78-1.69)	0.82(0.51-1.31)	0.94(0.57-1.54)	0.01(-0.25-0.27)	0(-0.26-0.25)
Other Government Worker	1.03(0.64-1.65)	1.18(0.73-1.93)	0.85(0.47-1.57)	1.05(0.56-1.97)	0.01(-0.32-0.34)	-0.05(-0.38-0.28)
Unemployed	0.94(0.68-1.3)	1.02(0.73-1.42)	0.77(0.51-1.17)	0.81(0.53-1.26)	0.03(-0.2-0.26)	0.02(-0.21-0.24)
Homemaker	0.96(0.74-1.26)	1.08(0.82-1.43)	0.89(0.64-1.24)	1.02(0.72-1.45)	0.09(-0.09-0.28)	0.02(-0.16-0.21)
Other, specify:	0.78(0.47-1.28)	0.89(0.53-1.5)	0.57(0.29-1.13)	0.59(0.28-1.24)	0.16(-0.19-0.51)	0.18(-0.17-0.53)
Don't know	0.76(0.27-2.14)	0.92(0.32-2.67)	0.45(0.09-2.16)	0.54(0.11-2.81)	0.42(-0.31-1.14)	0.17(-0.54-0.88)
**Father education (Ref=None)**						
Primary	1.04(0.63-1.74)	1.03(0.61-1.72)	0.94(0.50-1.77)	0.93(0.49-1.78)	0.09(-0.27-0.45)	0.14(-0.21-0.49)
Secondary	0.96(0.59-1.56)	1.03(0.63-1.69)	0.73(0.40-1.34)	0.82(0.44-1.53)	0.11(-0.23-0.45)	0.12(-0.21-0.46)
Technical/Vocational	1.04(0.37-2.92)	1.04(0.37-2.95)	1.31(0.40-4.33)	1.27(0.38-4.3)	-0.03(-0.76-0.7)	0.12(-0.59-0.83)
University/College	0.71(0.40-1.29)	0.8(0.43-1.46)	0.63(0.30-1.32)	0.8(0.37-1.73)	0.24(-0.18-0.66)	0.2(-0.21-0.62)
Don't know	0.92(0.57-1.47)	0.93(0.57-1.49)	0.87(0.49-1.56)	0.91(0.5-1.64)	0.13(-0.2-0.46)	0.17(-0.15-0.5)
**Mother's education (Ref=None)**						
Primary	0.94(0.65-1.35)	0.98(0.67-1.41)	1.06(0.67-1.69)	1.08(0.67-1.74)	-0.03(-0.28-0.23)	-0.02(-0.26-0.23)
Secondary	0.88(0.62-1.25)	0.97(0.68-1.4)	0.95(0.6-1.49)	1.04(0.65-1.67)	0.1(-0.15-0.34)	0.08(-0.16-0.33)
Technical/Vocational	0.41(0.15-1.16)	0.44(0.15-1.24)	0.17(0.02-1.35)	0.19(0.02-1.52)	1(0.31-1.69)	1(0.33-1.67)
University/College	0.87(0.54-1.4)	0.99(0.60-1.63)	0.75(0.39-1.42)	0.82(0.42-1.60)	0.09(-0.25-0.43)	0.11(-0.22-0.45)
Don't know	0.98(0.69-1.38)	1.06(0.75-1.51)	1.05(0.67-1.63)	1.12(0.71-1.77)	0.07(-0.17-0.31)	0.04(-0.19-0.28)
**Seen menstruation (Ref=No)**						
Yes	1.28(1.00-1.63)	1.18(0.91-1.53)	1.36(1.00-1.84)	1.22(0.88-1.68)	-0.13(-0.29-0.03)	-0.06(-0.23-0.1)
**Home garden available (Ref=No)**						
Yes	0.91(0.78-1.07)	0.91(0.77-1.07)	0.85(0.70-1.05)	0.84(0.68-1.04)	0.08(-0.03-0.19)	0.07(-0.04-0.18)
**Current cough (Ref=No)**						
yes	0.82(0.66-1.03)	0.84(0.66-1.06)	0.73*(0.54-0.99)	0.75(0.55-1.02)	0.14(-0.02-0.3)	0.14(-0.02-0.3)
**Cereals (Ref=< 1 per week)**						
1-6 per week	1.31(0.74-2.29)	1.47(0.83-2.61)	1.35(0.66-2.74)	1.53(0.74-3.15)	-0.05(-0.45-0.35)	-0.18(-0.57-0.21)
daily	1.03(0.6-1.75)	1.16(0.68-1.99)	0.96(0.49-1.88)	1.1(0.55-2.18)	0.08(-0.29-0.46)	-0.06(-0.42-0.31)
**Vegetables (Ref=< 1 per week)**						
1-6 per week	0.81(0.57-1.15)	0.78(0.55-1.11)	0.89(0.57-1.4)	0.78(0.49-1.24)	-0.01(-0.26-0.23)	0.06(-0.18-0.3)
daily	0.78(0.58-1.06)	0.78(0.57-1.05)	0.92(0.62-1.35)	0.86(0.58-1.28)	0(-0.21-0.21)	0.02(-0.18-0.23)
**Legumes (Ref=< 1 per week)**						
1-6 per week	0.93(0.76-1.13)	0.94(0.77-1.15)	0.98(0.76-1.26)	0.99(0.76-1.28)	0(-0.14-0.14)	-0.04(-0.17-0.1)
daily	0.92(0.75-1.13)	0.96(0.78-1.19)	0.94(0.72-1.22)	1.02(0.77-1.33)	0.1(-0.04-0.25)	0.02(-0.13-0.16)
**Fruits(Ref=< 1 per week)**						
1-6 per week	0.85(0.61-1.18)	0.83(0.59-1.17)	0.83(0.54-1.25)	0.78(0.51-1.19)	0.14(-0.09-0.38)	0.17(-0.06-0.4)
daily	0.76(0.57-1.01)	0.76(0.57-1.02)	0.73(0.52-1.04)	0.71(0.5-1.02)	0.15(-0.05-0.34)	0.16(-0.03-0.35)
**Meat(Ref=< 1 per week)**						
1-6 per week	0.92(0.78-1.09)	0.95(0.8-1.13)	0.93(0.75-1.15)	1.01(0.81-1.26)	0.04(-0.08-0.16)	-0.02(-0.14-0.09)
daily	0.94(0.69-1.28)	1.01(0.74-1.38)	0.7(0.46-1.08)	0.77(0.49-1.19)	0.19(-0.03-0.4)	0.06(-0.16-0.27)
**Eggs (Ref=< 1 per week)**						
Weekly	0.86(0.71-1.05)	0.89(0.73-1.09)	0.82(0.63-1.06)	0.84(0.65-1.1)	0.12(-0.02-0.26)	0.1(-0.04-0.24)
Daily	0.6(0.34-1.08)	0.58(0.32-1.05)	0.56(0.25-1.23)	0.6(0.27-1.36)	0.35(-0.06-0.76)	0.27(-0.13-0.67)
**Fish (Ref=< 1 per week)**						
1-6 per week	0.92(0.69-1.24)	0.93(0.69-1.25)	0.89(0.62-1.27)	0.89(0.61-1.3)	0.05(-0.15-0.26)	0.04(-0.16-0.24)
daily	0.78(0.59-1.04)	0.79(0.59-1.05)	0.72(0.5-1.01)	0.71(0.5-1.02)	0.11(-0.08-0.31)	0.08(-0.11-0.28)
**Milk (Ref=< 1 per week)**						
1-6 per week	0.91(0.69-1.2)	0.89(0.67-1.18)	0.9(0.63-1.26)	0.88(0.62-1.25)	0.02(-0.17-0.22)	0.03(-0.16-0.22)
daily	0.91(0.7-1.18)	0.9(0.69-1.17)	0.83(0.6-1.15)	0.81(0.58-1.12)	0.04(-0.14-0.22)	0.06(-0.11-0.24)
**Fats (Ref=< 1 per week)**						
1-6 per week	0.86(0.71-1.05)	0.89(0.73-1.09)	0.82(0.63-1.06)	0.84(0.65-1.1)	0.12(-0.02-0.26)	0.1(-0.04-0.24)
daily	0.6(0.34-1.08)	0.58(0.32-1.05)	0.56(0.25-1.23)	0.6(0.27-1.36)	0.35(-0.06-0.76)	0.27(-0.13-0.67)
**Beverages (Ref=< 1 per week)**						
1-6 per week	0.76(0.56-1.03)	0.81(0.6-1.1)	0.70(0.49-1.01)	0.77(0.53-1.12)	0.2(-0.01-0.41)	0.13(-0.08-0.33)
daily	0.74*(0.56-0.98)	0.8(0.6-1.06)	0.65*(0.46-0.91)	0.74(0.52-1.05)	0.27**(0.07-0.46)	0.17(-0.02-0.37)
**Sweets (Ref=< 1 per week)**						
1-6 per week	0.97(0.83-1.15)	0.99(0.84-1.17)	1.01(0.82-1.24)	1.03(0.83-1.28)	-0.07(-0.19-0.05)	-0.06(-0.18-0.05)
daily	1(0.71-1.42)	1.03(0.72-1.47)	1.08(0.7-1.67)	1.13(0.72-1.78)	0.03(-0.22-0.28)	0.05(-0.2-0.29)
**Tubers (Ref=< 1 per week)**						
1-6 per week	0.89(0.69-1.14)	0.87(0.67-1.13)	0.86(0.62-1.17)	0.82(0.59-1.13)	0.07(-0.11-0.25)	0.06(-0.12-0.23)
daily	0.84(0.66-1.08)	0.86(0.66-1.1)	0.75(0.55-1.03)	0.77(0.56-1.06)	0.17(-0.01-0.35)	0.12(-0.06-0.29)
**Tamarind (Ref=No)**						
Yes	0.95(0.77-1.18)	0.93(0.75-1.16)	0.96(0.73-1.26)	0.94(0.71-1.25)	-0.05(-0.2-0.1)	-0.01(-0.15-0.14)
**Beans (Ref=No)**						
Yes	0.95(0.8-1.13)	0.95(0.8-1.14)	0.89(0.71-1.12)	0.92(0.73-1.17)	0.1(-0.03-0.22)	0.06(-0.06-0.18)
**Spinach (Ref=No)**						
Yes	1.32(1.06-1.66)	1.32(1.05-1.66)	1.22(0.91-1.62)	1.23(0.92-1.65)	-0.05(-0.21-0.1)	-0.06(-0.22-0.09)
**Pumpkin leaves (Ref=No)**						
Yes	0.98(0.8-1.2)	0.93(0.76-1.15)	0.91(0.7-1.19)	0.86(0.65-1.13)	0.02(-0.13-0.16)	0.04(-0.11-0.18)
**Pumpkin (Ref=No)**						
Yes	1.12(0.94-1.32)	1.11(0.93-1.32)	1.07(0.87-1.33)	1.08(0.87-1.35)	-0.03(-0.15-0.09)	-0.06(-0.17-0.06)
**Beef (Ref=No)**						
Yes	0.95(0.81-1.11)	1(0.85-1.17)	0.87(0.71-1.07)	0.94(0.76-1.16)	0.07(-0.04-0.18)	-0.02(-0.13-0.09)
**Pork (Ref=No)**						
Yes	2.43(1.08-5.49)	2.55(1.11-5.86)	1.81(0.65-5.01)	2.15(0.75-6.13)	-0.46(-0.98-0.06)	-0.55*(-1.06--0.04)
**Liver (Ref=No)**						
Yes	0.82(0.67-1.01)	0.87(0.71-1.08)	0.71*(0.54-0.94)	0.76(0.57-1)	0.1(-0.05-0.24)	0.02(-0.12-0.16)
**Chicken (Ref=No)**						
Yes	0.84(0.7-1.01)	0.88(0.73-1.06)	0.81(0.64-1.02)	0.84(0.66-1.06)	0.12(-0.01-0.25)	0.06(-0.07-0.19)
**Fried fish (Ref=No)**						
Yes	0.82(0.62-1.09)	0.85(0.64-1.14)	0.89(0.62-1.27)	0.93(0.64-1.36)	0.06(-0.14-0.26)	0.03(-0.16-0.23)
**Fresh fish (Ref=No)**						
Yes	0.97(0.78-1.21)	0.97(0.77-1.22)	0.86(0.65-1.12)	0.87(0.65-1.15)	0.02(-0.14-0.17)	-0.01(-0.16-0.14)
**Sardines (Ref=No)**						
Yes	0.86(0.73-1.02)	0.88(0.74-1.05)	0.83(0.67-1.03)	0.84(0.67-1.05)	0.05(-0.07-0.17)	0(-0.12-0.12)
**Dried fish (Ref=No)**						
Yes	1.02(0.86-1.21)	1(0.84-1.19)	1.04(0.83-1.29)	1.05(0.84-1.31)	0.01(-0.11-0.13)	-0.02(-0.14-0.1)
**Water Source (Ref=Piped into house /bottled water)**						
Piped into neighbourhood	1.03(0.85-1.25)	0.97(0.79-1.19)	1.06(0.83-1.36)	0.98(0.76-1.27)	0.00(-0.14-0.14)	0.04(-0.1-0.17)
Well or surface water	1.06(0.86-1.31)	1.04(0.84-1.29)	1.03(0.78-1.35)	0.97(0.73-1.28)	-0.01(-0.16-0.14)	-0.01(-0.16-0.13)
**Water Treatment method (Ref=Boil)**						
Add bleach/chlorine	0.68*(0.48-0.96)	0.68*(0.48-0.97)	0.66(0.42-1.03)	0.68(0.43-1.07)	0.21(-0.03-0.45)	0.19(-0.05-0.43)
Do not treat water	0.9(0.71-1.14)	0.88(0.69-1.13)	0.92(0.68-1.24)	0.9(0.66-1.22)	0.05(-0.12-0.22)	0.04(-0.12-0.21)
Other, specify	0.64(0.36-1.14)	0.65(0.36-1.18)	0.55(0.25-1.22)	0.54(0.24-1.22)	0.18(-0.22-0.59)	0.2(-0.2-0.6)
**Toilet type (Ref=Flush or pour flush toilet)**						
Pit toilet/latrine/no facility/bush/filed	1.08(0.92-1.28)	1(0.84-1.2)	1.23(0.99-1.51)	1.12(0.89-1.41)	-0.08(-0.2-0.04)	-0.05(-0.17-0.07)
**Shared toilet (Ref=No)**						
Yes	1.19(0.82-1.73)	1.21(0.83-1.78)	1.57*(1.01-2.42)	1.68*(1.07-2.64)	-0.27*(-0.53--0.01)	-0.30*(-0.56--0.04)
**Brushing teeth (Ref=No)**						
Once per day	1.57(0.62-3.93)	1.42(0.56-3.6)	1.27(0.4-4.05)	1.18(0.35-3.9)	0(-0.64-0.65)	0.14(-0.49-0.76)
Twice or more per day	1.58(0.63-3.95)	1.41(0.55-3.57)	1.38(0.44-4.37)	1.22(0.37-4.02)	-0.1(-0.74-0.54)	0.11(-0.52-0.73)
**Dentist visits in the past year (Ref=No)**						
Yes	1.14(0.86-1.5)	1.13(0.85-1.51)	1.06(0.75-1.51)	1.08(0.75-1.57)	-0.03(-0.22-0.17)	-0.03(-0.22-0.16)
**Handwashing before eating(Ref=Never/Rarely)**						
Sometimes	0.93(0.66-1.31)	0.89(0.63-1.26)	1.22(0.78-1.93)	1.14(0.72-1.82)	-0.1(-0.34-0.14)	-0.02(-0.26-0.21)
Most of the time	1.16(0.86-1.56)	1.12(0.83-1.51)	1.37(0.92-2.05)	1.28(0.84-1.93)	-0.14(-0.35-0.07)	-0.06(-0.26-0.15)
Always	1.14(0.86-1.51)	1.12(0.84-1.5)	1.43(0.97-2.1)	1.35(0.91-2.01)	-0.17(-0.37-0.03)	-0.08(-0.28-0.11)
**Handwashing after toilet (Ref=Never/Rarely)**						
Sometimes	1.11(0.85-1.45)	1.1(0.84-1.44)	1.05(0.74-1.49)	1.04(0.73-1.48)	-0.04(-0.22-0.15)	0(-0.18-0.18)
Most of the time	1(0.78-1.28)	1(0.77-1.28)	1.15(0.83-1.58)	1.16(0.83-1.61)	-0.05(-0.23-0.13)	0(-0.17-0.18)
Always	1.13(0.9-1.42)	1.13(0.9-1.43)	1.27(0.94-1.71)	1.29(0.95-1.76)	-0.15(-0.31-0.02)	-0.1(-0.26-0.06)
**Handwashing method (Ref=Proper)**						
Improper	0.98(0.82-1.17)	0.99(0.82-1.19)	1.01(0.81-1.27)	1.04(0.82-1.31)	0.05(-0.08-0.17)	0.01(-0.11-0.14)

Odds ratios and 95% confidence intervals are derived from logistic regression (binary anemia outcomes) and linear regression (continuous hemoglobin) models. Adjusted models control for sex, age, wealth, BMI, malaria, and stunting. *p<0.05; **p<0.01; ***p<0.001

Adolescents who were stunted were found to have higher odds of any anemia (OR-1.38; 95% CI: 1.06,1.81) and moderate severe anemia (aOR = 1.45; 95%CI: 1.05,2.02) while their level of hemoglobin were lower only in the adjusted model (b=-0.44; CI: -0.74-0.15). Similarly, adolescents who were underweight had higher odds of moderate/severe anemia in both crude and adjusted models (aOR = 0.58; CI: 0.32–0.95) compared to adolescents who had normal weight. There was no difference between those who were overweight/obese and those who had normal weight.

Adolescents who consumed more beverages had lower odds of any anemia (aOR = 0.74; CI: 0.56–0.98), moderate/severe (aOR = 0.65; CI: 0.46–0.91) as well as lower levels of hemoglobin (b = 0.27; CI: 0.07–0.46); however this relationship loses significance after controlling for socio-demographic factors. Among different foods, those who consumed liver had lower odds of moderate/severe anemia (aOR = 0.71; CI: 0.54–0.94) compared to those who didn’t.

On investigating WASH variables, adolescents who reported treating their drinking water by adding bleach/chlorine had lower odds of anemia (aOR = 0.68; CI: 0.48–0.96) compared to those who boiled. No difference was observed among those who reported not treating their water vs. those who boiled. Those who shared toilet facility had higher odds of moderate/severe anemia (aOR = 1.57; CI:1.01–2.42) and lower levels of hemoglobin (b = 00.27; CI: -0.53 - -0.01) in both crude and adjusted models compared to those who didn’t share.

Adolescent girls who had experienced menarche had higher odds of moderate/severe anemia than those who had not in the crude model (aOR = 1.36; CI:1.00-1.84) however the relationship was not significant in the adjusted model (*p*-value 0.05).

## Discussion

In this cross-sectional survey of school-going adolescents in Zanzibar, we found that slightly over 50% of the population had hemoglobin levels below the cutoff for anemia. Though most anemia cases were mild, over 20% of the population had moderate or severe anemia. While we cannot compare our results to previous findings on the prevalence of anemia in this particular sub-population due to the inexistence of such studies, the high prevalence of anemia among adolescents is consistent with findings from the TDHS showing high anemia levels in Zanzibar among children under five years of age (65.4% in 2015) and women of reproductive age (60% in 2015) [7, 19]. Compared to women and children under five, the prevalence of anemia in adolescents is slightly lower, though still concerningly high. In fact, adolescents in Zanzibar have the highest reported prevalence of anemia among adolescents across the few low- and middle- income countries (LMICs) with data on adolescents to date; prior to this study, reported prevalence from surveys that assessed hemoglobin levels ranged from 27% among adolescent secondary school girls in Ethiopia [20] to 49% among adolescent girls in India [21].

Odds of anemia in our sample were higher among females, adolescents with stunting, and those who used shared toilets (compared to personal household toilets). Females who had experienced menarche also had higher odds of moderate or severe anemia than those who had not yet starting menstruating, demonstrating the likely importance of blood losses contributing to low hemoglobin levels and to the progression of anemia beyond mild cases [22, 23]. However, this relationship was not significant in our study after adjusting for socio-demographic factors. In addition to fitting with previous findings that anemia status is higher among females globally [24], including adolescents [25], these findings on differential anemia status by sex are consistent with findings from a study we conducted among a similar population of adolescents (aged 10–19) in Burkina Faso. There, though females were overall less likely to have anemia than males, they were also more likely to have moderate or severe anemia, had lower hemoglobin levels, and had higher odds of anemia if they had experienced menarche [26]. In both Zanzibar and Burkina Faso, even though females have higher odds of anemia than males, the prevalence of anemia among adolescent males is still high, and merits action.

The association of stunting with anemia as found in our sample has also been demonstrated previously [27, 28], including in our study of adolescents in Burkina Faso [26], and a study of anemia among adolescents in India [25]. One key hypothesis about this correlation is that stunting and anemia are caused by similar environmental and dietary conditions, thus adolescents (and others) who are stunted are also more likely to develop anemia due to shared risk factors. Our results support this hypothesis as the factors identified in the analysis as being associated with anemia, including poor wealth and hygiene indicators (notably, sharing toilet facilities) have previously been shown to be associated with concurrent stunting and anemia [25, 28, 29]. Dietary factors are also likely associated with both stunting and anemia [28, 30], though our study results have not demonstrated as such beyond the association between liver consumption and lowered anemia odds, potentially due to the limited nature of our dietary data collection which allow for analysis of only food frequency but not intake of food quantities.

These findings support the idea that in addressing anemia, it is important to focus on the cycle between infectious disease, inflammation, and anemia, in addition to dietary factors that may influence nutritional status. Infection (with helminths or other parasites) due to poor hygiene conditions has been strongly linked to anemia among school children [31–33], and inflammation has been identified as an important contributor to anemia among school-going adolescents [34]. These factors are directly related to water, sanitation, and hygiene, and while handwashing was not significantly associated with reduced risk of anemia in our study, access to private sanitation, treatment of water, and wealth, which is a proxy indicator for overall household conditions, were all significantly associated with anemia. Improved access to sanitation has recently been shown to lower anemia risk among women of childbearing age in southern Africa [35], as has improved access to clean water among pre-school children in Ethiopia [36]. The observed positive relationship between using shared toilets and anemia among adolescents in our sample, as well as that of lowered odds of anemia with use of bleach or chlorine to treat water provide further evidence of this relationship between water, sanitation and hygiene and anemia, and the importance of programs that address these issues.

The limitations of this study include the fact that it was conducted in only two out of the seven districts in Zanzibar, so we do not have representation from all sub-regions of Zanzibar, and that it was conducted solely among school-going adolescents, which may limit generalizability of the results. In addition, we did not collect dietary quantity data, and formulate conclusions related to diet based only relative food frequencies. Nevertheless, the study included a large sample size of adolescents in Zanzibar and is to date the only study to have investigated the prevalence of anemia among adolescents in Zanzibar, and the factors associated with anemia in the same population.

## Conclusions

The factors found to be associated with anemia among adolescents in Zanzibar align well with factors associated with anemia among pregnant and non-pregnant women of reproductive age across many LMICs, as well as factors associated with anemia among adolescents in similar low- and middle-income country contexts. The identification of these factors highlights the importance of comprehensive programs that address both dietary and environmental (water, sanitation and hygiene) factors, and that aim to bolster household wealth and socioeconomic status to improve household and dietary conditions. In addition, adolescent males should not be overlooked in programming, given their important burden of anemia. This evidence should be used urgently to guide policy and programs aimed at lowering the high burden of anemia found among adolescents in Zanzibar.

### Supplementary Information

**Additional file 1.** Supplementary Materials for Factors associated with anemia among school-going adolescents in Zanzibar

## Data Availability

The datasets used and analyzed during the current study are available from the corresponding author on reasonable request.

## Abbreviations

BMI: Body mass index
HDDS: Household Diet Diversity Score
IFA: Iron + folic acid
LMICs: Low- and middle- income countries
MMS: Multiple micronutrient supplement
TDHS: Tanzania Demographic and Health Survey
WASH: Water, sanitation and hygiene
WHO: World Health Organization
