# Supplementary material for: Factors associated with anemia among school-going adolescents aged 10–17 years in Zanzibar, Tanzania: a cross sectional study
Source: BMC Public Health. 2023 Sep 18;23:1814. doi: 10.1186/s12889-023-16611-w (PMC10508009; doi:10.1186/s12889-023-16611-w)
Supplement: Supplementary file 1 — Additional file 1. Supplementary Materials for Factors associated with anemia among school-going adolescents in Zanzibar [file 12889_2023_16611_MOESM1_ESM.docx]

Supplementary Materials for Factors associated with anemia among school-going adolescents in Zanzibar

| **Supplementary Table 1. Minimum detectable effect sizes for factors potentially associated with anemia among adolescents taking part in SAMIA trial, Zanzibar, 2022** | |  |
| --- | --- | --- |
|  |  |  |
|  | **Effect size^ (percentage points)** |  |
| **Socio-demographic and biological factors** |  |  |
| Age | 17 |  |
| Sex | 6 |  |
| Currently lives with | 14 |  |
| Number of siblings | 14 |  |
| Father's occupation | 11 |  |
| Mother's occupation | 18 |  |
| Father's education | 19 |  |
| Mother's education | 21 |  |
| SES Quintile | 6 |  |
| Seen menstruation | 9 |  |
| Home garden available | 6 |  |
| Current cough | 8 |  |
| Malaria diagnosis | 16 |  |
| Stunting | 9 |  |
| BMI for age z-score category | 12 |  |
| Household diet diversity score | 5 |  |
| **Food Groups** |  |  |
| Cereals | 16 |  |
| Vegetables | 10 |  |
| Legumes | 6 |  |
| Fruits | 8 |  |
| Meat | 8 |  |
| Eggs | 17 |  |
| Fish | 8 |  |
| Milk | 7 |  |
| Fats | 17 |  |
| Beverages | 8 |  |
| Sweets | 10 |  |
| Tubers | 7 |  |
| **Iron-rich foods** |  |  |
| Tamarind | 8 |  |
| Beans | 6 |  |
| Spinach | 8 |  |
| Pumpkin leaves | 7 |  |
| Pumpkin | 6 |  |
| Beef | 6 |  |
| Pork | 32 |  |
| Liver | 7 |  |
| Chicken | 6 |  |
| Fried fish | 10 |  |
| Fresh fish | 7 |  |
| Sardines | 6 |  |
| Dried fish | 6 |  |
| **WASH** |  |  |
| Water source | 6 |  |
| Household treats water | 6 |  |
| Water treatment method | 8 |  |
| Toilet type | 6 |  |
| Shared toilet | 13 |  |
| Brushing teeth | 24 |  |
| Dentist visits in past year | 10 |  |
| Handwashing before eating | 7 |  |
| Handwashing after toilet | 6 |  |
| Handwashing method | 6 |  |
| Effect sizes show the smallest effect size that can be detected given our sample sizes based on a 5% alpha level and 80% power and were calculated using Stata 16.1 *power* commands. For variables with more than 2 groups, the smallest and largest sample sizes per group were used to calculate the minimum detectable effect size. The expected prevalence of anemia was set as 53%, as this was the baseline prevalence in the present sample. | |  |
